# Application of the Oval Femoral Tunnel Technique in Anatomical Single‐Bundle Anterior Cruciate Ligament Reconstruction: Fujian Union Technique

**DOI:** 10.1002/atn2.70271

**Published:** 2026-10-08

**Authors:** Weihuang Lin, Chengjie Lian, Chengjian Wu, Zhi Chen, Zhengru Wu, Hua Zhang

**Affiliations:** ^1^ Department of Orthopaedics, Sports Injury Division Fujian Medical University Union Hospital Fuzhou China

## Abstract

Anterior cruciate ligament (ACL) reconstruction aims to restore both the anatomical footprint and the biomechanical stability of the knee joint. However, traditional femoral tunnel techniques, typically round in shape, often fail to accurately replicate the oval femoral insertion of the ACL. This discrepancy can compromise rotational stability and hinder graft integration. The oval femoral tunnel technique addresses this issue by creating a tunnel that more closely matches the anatomical dimensions of the ACL footprint. This article describes the oval femoral tunnel dilator and its accompanying instruments, detailing their application as a surgical technique for ACL reconstruction.

## Graphical abstract

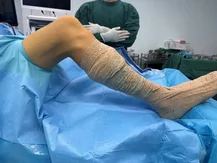

**VIDEO 1:** Surgical demonstration of anatomical single‐bundle anterior cruciate ligament reconstruction using the oval femoral tunnel technique in a right knee. The patient is placed in the supine position. The video begins with the preparation of a 4‐strand hamstring autograft, which is passed through an adjustable‐loop suspensory device. The graft's dimensions are precisely measured using a specialized oval sizer. Viewing from the anterolateral portal, diagnostic arthroscopy confirms the anterior cruciate ligament rupture. Viewing through the central patellar tendon observation portal, the native anterior cruciate ligament femoral footprint is identified, and its long axis is marked using a radiofrequency wand. A guide pin is then inserted precisely at the center of the footprint. The core step of the technique is subsequently shown: To maximize cancellous bone preservation, no pilot drill is used. Instead, an appropriately sized oval tunnel dilator is introduced through the anteromedial portal and impacted directly over the guide pin. The surgeon uses gentle, controlled mallet taps to advance the dilator to a predetermined depth of 20 mm, ensuring its long axis strictly aligns with the marked native footprint to prevent iatrogenic condyle fractures. Following dilator extraction, a 4.5‐mm cannulated drill is used to create the cortical exit tunnel. Attention is then turned to the tibia. Viewing from the anterolateral portal, a tibial guide is positioned at the anatomical footprint, and a conventional tibial tunnel is reamed. The graft is passed through the tibial tunnel into the oval femoral socket. The adjustable loop is flipped over the lateral femoral cortex for secure suspensory fixation. Finally, tibial fixation is achieved using an interference screw supplemented by a U‐staple under proper tension. The video concludes with a postoperative three‐dimensional computed tomography reconstruction (medial view of the lateral femoral condyle), which confirms the accurate oval morphology and anatomical placement of the femoral tunnel. Video content can be viewed at https://doi.org/10.1002/atn2.70271.

Anterior cruciate ligament (ACL) injury is a common knee joint sports‐related trauma, and the ACL plays a critical role in maintaining knee stability. For patients with ACL injury or rupture, arthroscopic anatomic ACL reconstruction is a commonly recommended treatment option. However, there is ongoing debate regarding the technical details of ACL reconstruction, particularly in relation to the femoral tunnel creation. Traditionally, round femoral tunnels have been used in single‐bundle reconstruction; however, the ACL femoral footprint is an oval, flat‐shaped structure, making it difficult for round tunnels to fully replicate the anatomical footprint. Recent literature continues to emphasize that precise replication of the native ACL footprint morphology is paramount for optimizing postoperative knee kinematics and restoring true rotational stability.^1^, ^2^ In recent years, the oval tunnel technique has gained attention for its ability to better match the anatomical morphology of the ACL. Our team designed and patented an oval tunnel dilator and its accompanying instruments (Chinese Patent No. ZL201621452946.2) based on the anatomical characteristics of the Chinese population's knee joints. This instrument set features a tendon measuring device and a corresponding extractor that differ from previously reported designs, and it has shown favorable clinical outcomes.^3^, ^4^ This paper provides a detailed description of the surgical steps, advantages, and precautions of using this oval tunnel expander for ACL anatomic single‐bundle reconstruction.

## SURGICAL TECHNIQUE

### Patient Positioning and Preparation

The patient is placed in the supine position under either epidural or general anesthesia. A tourniquet is not applied to the proximal femur. A lower limb support is positioned to allow the knee joint to flex to 80° to 90°, and a lateral support is placed on the proximal femur to facilitate better knee external rotation during the procedure (Figure 1). Following routine disinfection and draping, a standard anterolateral portal is established, followed by the creation of a standard anteromedial portal and an observation portal between the patellar tendons (Figure 2). Diagnostic arthroscopy is performed to confirm ACL rupture and associated injuries (Figure 3).

**FIGURE 1:**
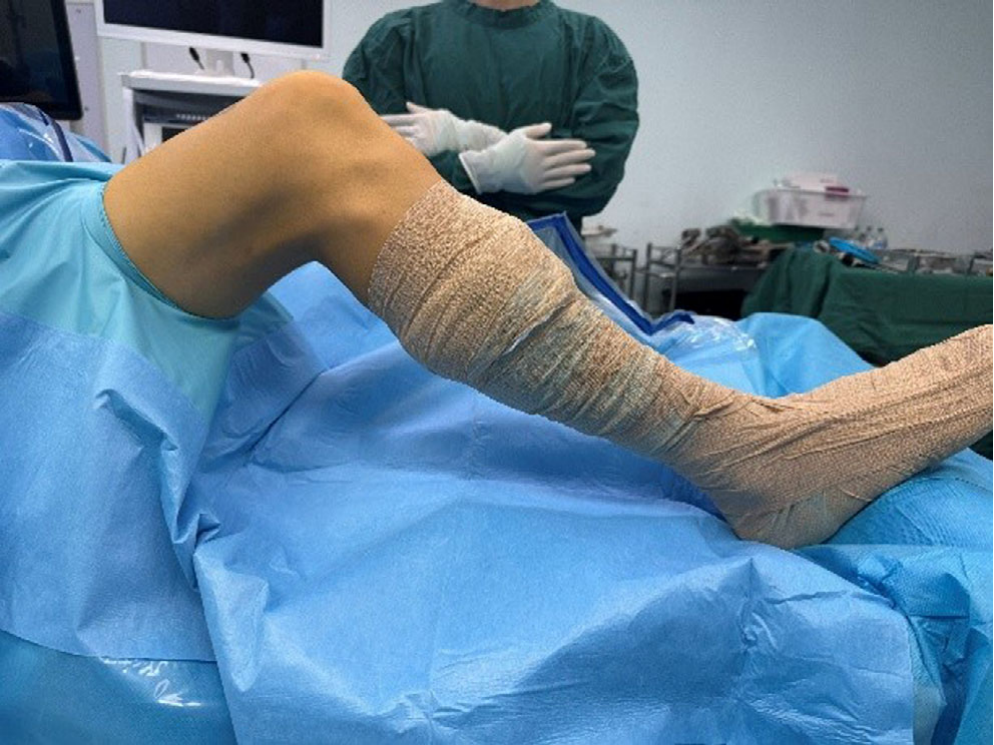
Right knee: The entire lower limb is positioned at the end of the bed, with a footrest placed to allow the knee joint to flex to 80°‐90°. A lateral support is placed to facilitate better external rotation of the knee during the procedure.

**FIGURE 2:**
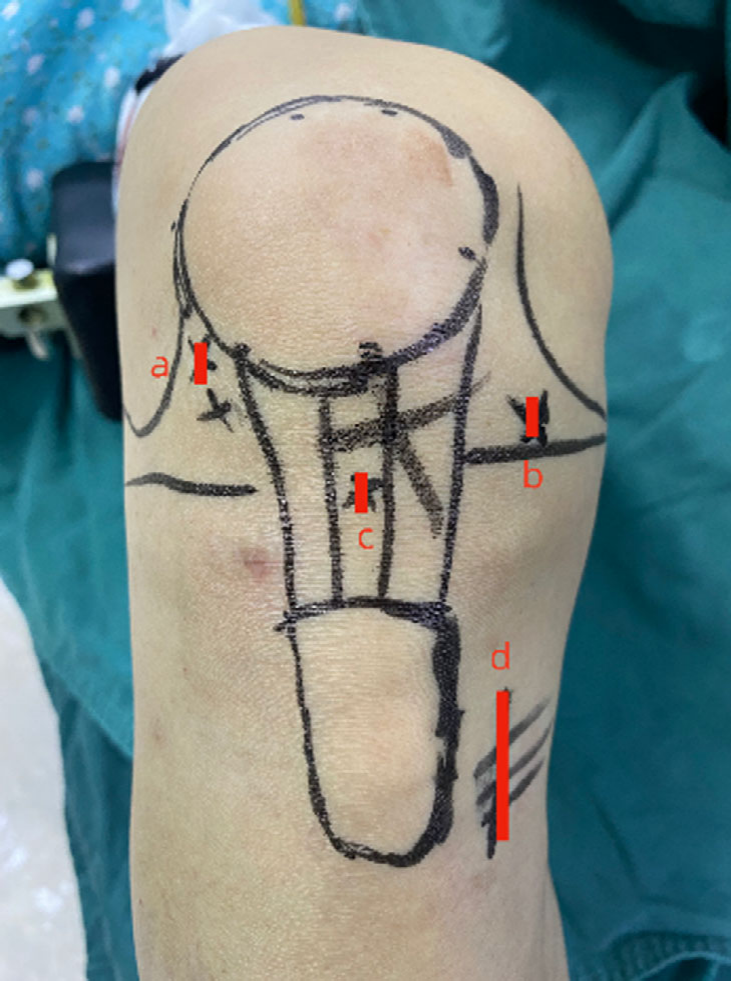
Clinical photograph of the right knee showing the surgical incisions. (a) Anterolateral portal for initial arthroscopic viewing. (b) Anteromedial portal for instrumentation and femoral tunnel drilling. (c) Central observation portal located through the patellar tendon. (d) A 3‐cm longitudinal incision placed medial to the tibial tuberosity for autograft harvesting.

**FIGURE 3:**
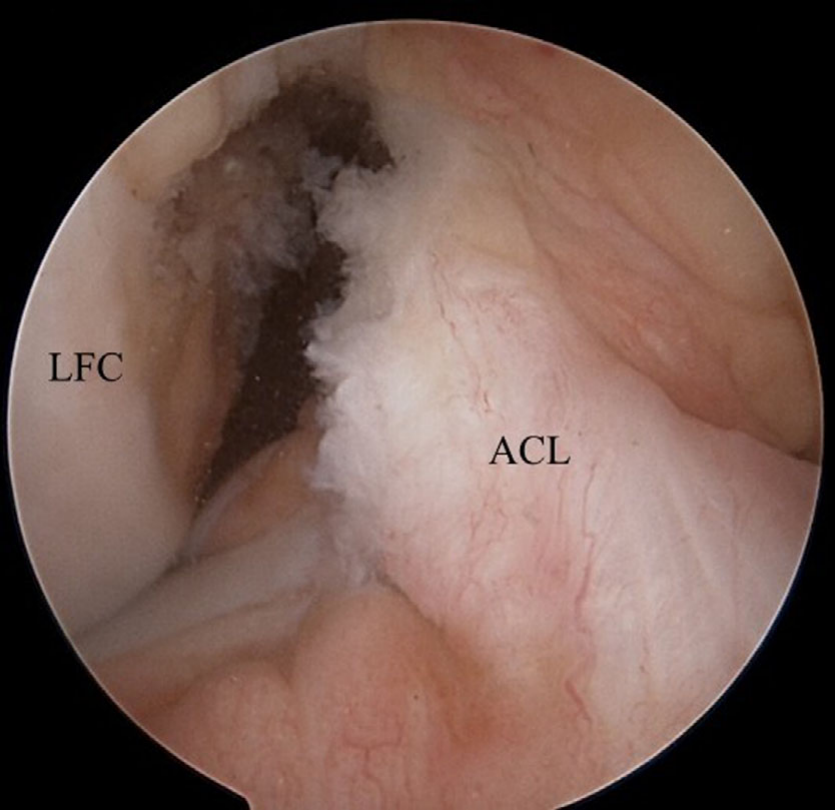
Right knee: A standard anterolateral portal is established, followed by the creation of a standard midpatellar portal under arthroscopic visualization. Diagnostic arthroscopy is performed to confirm ACL rupture from LFC and associated lesions. (ACL, anterior cruciate ligament; LFC, lateral femoral condyle.)

### Graft Harvesting and Preparation

A longitudinal incision of approximately 3 cm is made 1 cm medial to the tibial tuberosity. The subcutaneous tissue and deep fascia are incised, and the pes anserinus insertion is exposed. Using a right‐angle clamp, the semitendinosus and gracilis tendons are fully mobilized. The tendon branches are separated using tissue scissors. With the knee flexed to 90°, the tendons of the gracilis and semitendinosus muscles are harvested along the direction of their fibers using a tendon stripper. The fat tissue on the surface of the tendons and the muscle tissue attached to the tendon ends are removed. The tendons are reinforced with high‐strength suture (Non‐absorbable surgical suture, Demey Company). The 2 tendon grafts are folded to form a 4‐strand graft after being passed through the adjustable loop (FixButton with Loop, Demey Company) (Figure 4). The tendon grafts are then passed through an oval tunnel gauge to select the corresponding oval tunnel expander (Figure 5). The sizes of the tunnel gauge and expander correspond to one another and include various dimensions, such as 12 × 8 mm, 12 × 7 mm, 11 × 7 mm, 10 × 7 mm, 11 × 6 mm, 10 × 6 mm, 9 × 6 mm, and 8 × 6 mm (Figures 6 and 7). The diameter of the graft is measured; if the diameter measured with the round gauge does not exceed 7 mm (Figure 8), flat sutures are added to increase the diameter. The graft is then wrapped in saline‐soaked gauze and prepared for use.

**FIGURE 4:**
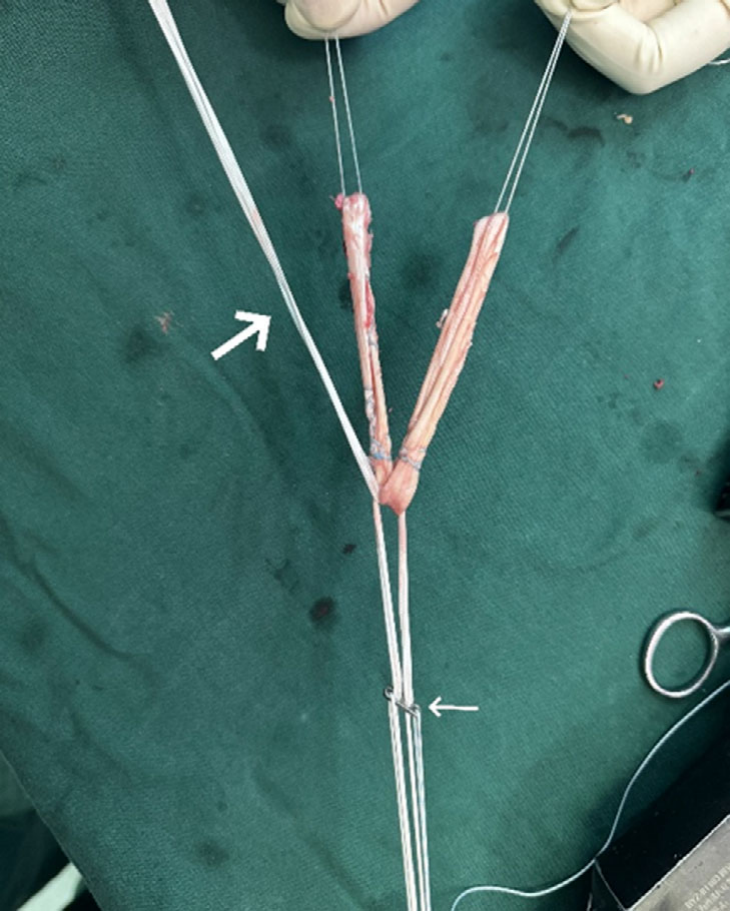
The grafts are reinforced with high‐strength suture (thick white arrow). The 2 tendon grafts are passed through a small adjustable loop (thick white arrow), and flat sutures are added if the graft diameter is smaller.

**FIGURE 5:**
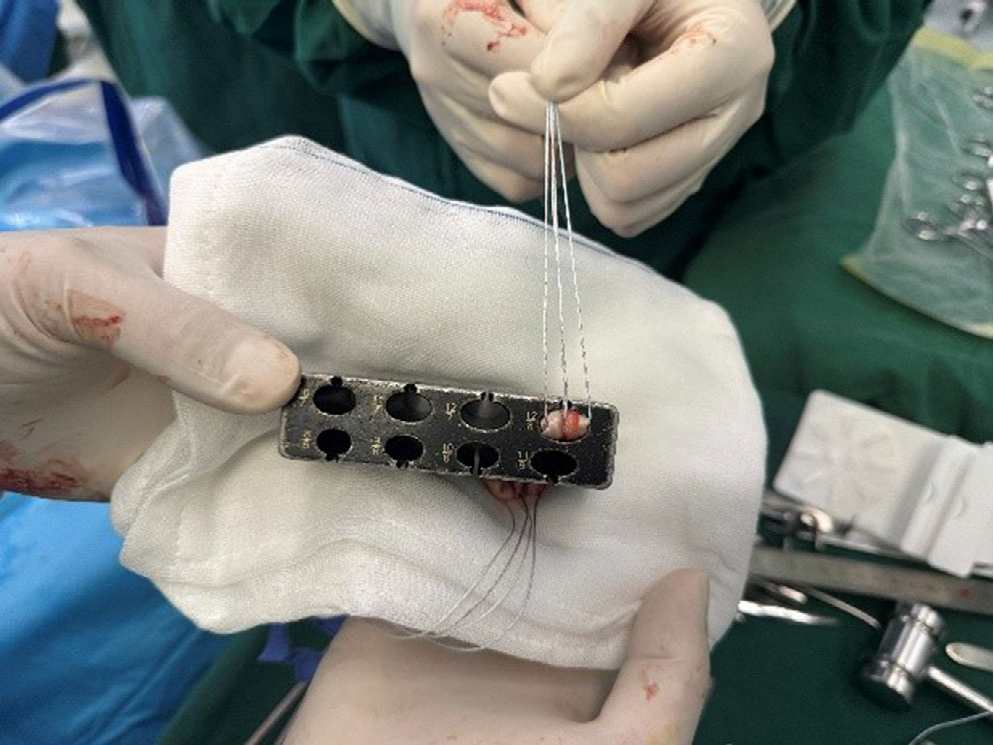
Measuring the graft size using the oval tunnel gauge.

**FIGURE 6:**
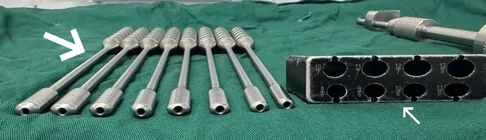
Frontal view of the oval tunnel gauge (thin white arrow) and expander (thick white arrow), with size specifications of 12 × 8 mm, 12 × 7 mm, 11 × 7 mm, 10 × 7 mm, 11 × 6 mm, 10 × 6 mm, 9 × 6 mm, and 8 × 6 mm.

**FIGURE 7:**
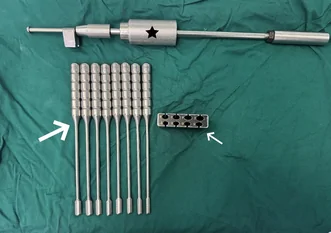
Top view of the oval tunnel instrument set, including a tendon gauge (thin white arrow), an extractor (black star), and 8 different sizes of tunnel expanders (thick white arrow).

**FIGURE 8:**
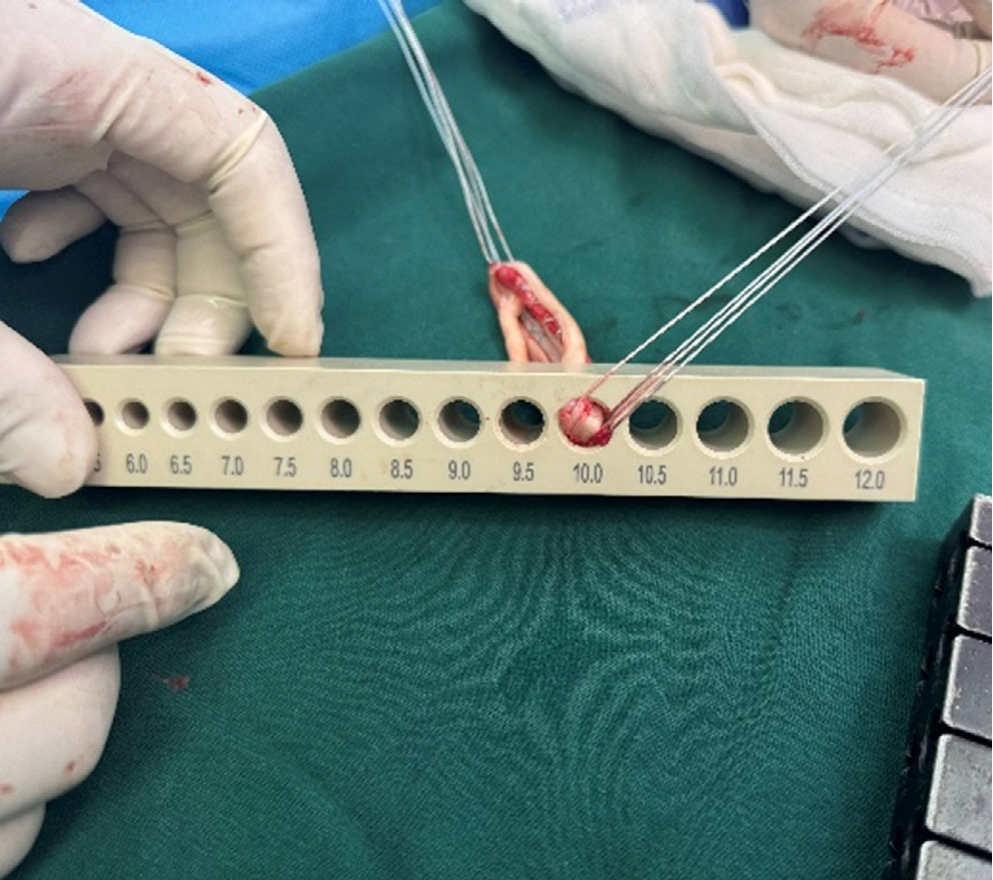
Measuring the graft size using the round tunnel gauge.

### Creation of the Oval Femoral Tunnel

Through the anteromedial portal, the intercondylar notch of the femur is cleaned using a reamer to expose the femoral footprint of the ACL. A plasma knife (Disposable radiofrequency ablation electrode needle, Bangshi Medical Technology) is then used to clear the remnants of the ACL and other soft tissue at the femoral footprint. Through the midpatellar tendon portal, the ACL femoral footprint is identified. Using anatomical landmarks such as resident ridge and bifurcate ridge, the center of the footprint is located. The plasma knife is used to mark the long‐axis direction of the ACL footprint along the femoral footprint imprint. A guidewire is inserted at the midpoint of the ACL femoral footprint, and the corresponding size of the oval tunnel expander is selected based on the graft diameter and the oval gauge.

The knee is then hyperflexed. To maximize bone preservation and reduce tunnel enlargement, no pilot drill is used prior to the insertion of the oval expander. Instead, the expander is impacted directly over the guidewire through the anteromedial portal. To mitigate the potential risk of iatrogenic lateral femoral condyle fracture associated with this direct impaction, it is imperative that the surgeon advances the dilator using gentle, controlled taps with a bone mallet rather than forceful blows. Furthermore, the expander must remain strictly collinear with the guidewire trajectory throughout the entire 20‐mm advancement process. Afterward, the oval tunnel expander is removed using an extractor. A 4.5‐mm hollow drill is then used along the guidewire to drill through the lateral femoral cortex, creating the fine tunnel. The depth of the tunnel is measured, and the total tunnel depth is marked on the loop. A traction suture is passed through the femoral tunnel using the guidewire for future use. The arthroscopic procedure is shown in Figure 9, and the ex vivo procedure is shown in Figure 10. The overall schematic diagram is shown in Figure 11.

**FIGURE 9:**
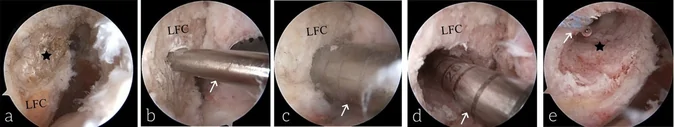
Arthroscopic view from the central observation portal of the process of establishing the oval femoral tunnel in the right knee. (A) The long‐axis direction of the ACL femoral footprint (black star) is marked using a plasma knife through the anteromedial portal; (B) the femoral guidewire (white arrow) entry point is drilled at the midpoint of the ACL footprint; (C) the oval tunnel expander (white arrow) is introduced through the anteromedial portal and gently advanced along the guidewire using a mallet; (D) the insertion depth of the oval tunnel expander is confirmed at 20 mm; and (E) final view of the completed oval femoral tunnel (black star), showing a morphologically accurate replication of the footprint, with the traction suture (white arrow) visible in the upper lateral aspect. (ACL, anterior cruciate ligament; LFC, lateral femoral condyle.)

**FIGURE 10:**
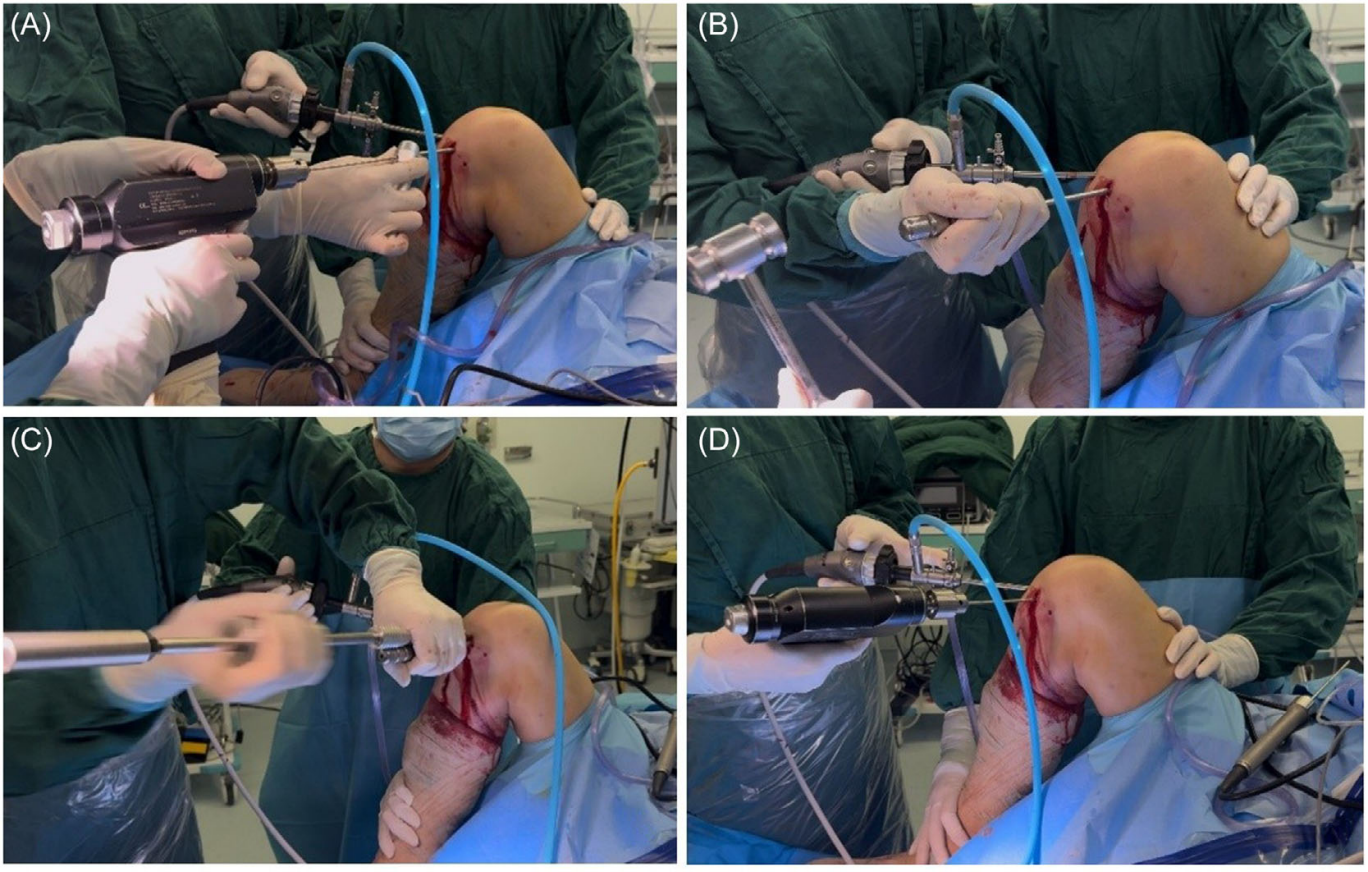
Femoral tunnel establishment in the right knee, as seen from the patient's left side. (A) The femoral guidewire is drilled through the anteromedial portal; (B) the oval tunnel expander is gently struck with a bone mallet to expand the tunnel; (C) the oval tunnel expander is removed using an extractor; and (D) a 4.5‐mm hollow drill is used along the guidewire to drill through the lateral femoral cortex, creating the fine tunnel.

**FIGURE 11:**
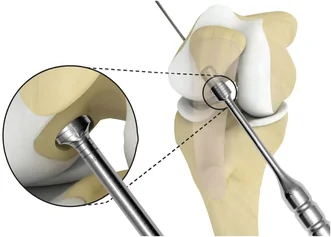
The schematic diagram of the oval tunnel expander being inserted into the femoral tunnel from the medial view of the right knee: The knee is then hyperflexed, and through the anteromedial portal, the expander is gently hammered along the direction of the guidewire to a depth of 20 mm.

### Tibial Tunnel Creation

Through the anterolateral portal, the knee joint is examined arthroscopically to identify the tibial footprint of the ACL. The tibial tunnel entry point is then marked using a radiofrequency device. The angle of the tibial tunnel guide (DePuy Mitek, Raynham, MA) is set at 55°, and the guide is anchored at the center of the ACL remnant through the anteromedial incision. For patients without an ACL tibial remnant, the tibial tunnel positioning is based on the anatomical study of the ACL by Petersen et al. The tibial guidewire is drilled through the guide, and a hollow reamer of appropriate size is selected based on the graft diameter. The reamer is advanced along the guidewire to prepare the tibial tunnel (Figure 12).

**FIGURE 12:**
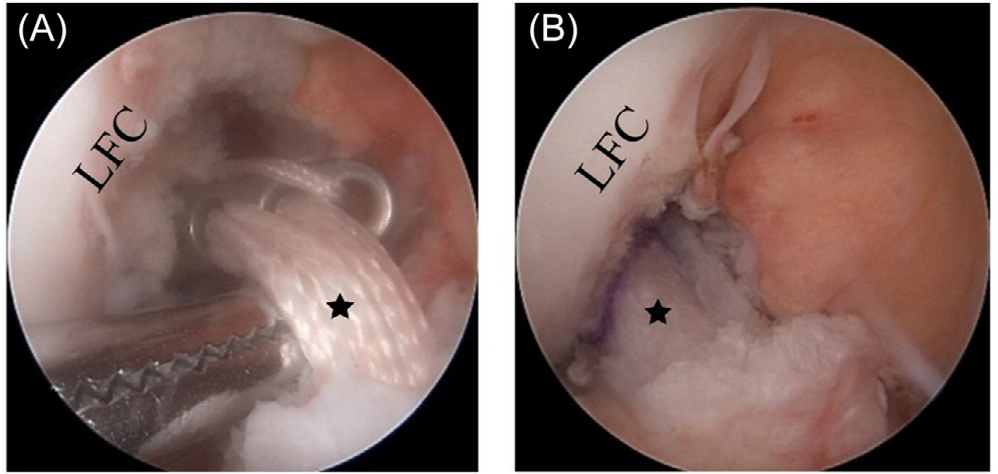
Arthroscopic intraoperative view of the right knee, viewing from the central patellar tendon observation portal, showing graft passage and final fixation. (A) The 4‐strand autograft (black star) is passed through the tibial tunnel and pulled into the oval femoral tunnel using the adjustable‐loop device. (B) Final graft (black star) positioning shows excellent tension and anatomical footprint restoration, with no evidence of impingement in the intercondylar notch during full knee range of motion. (LFC, lateral femoral condyle.)

### Graft Insertion and Fixation

After the bone tunnels are prepared, the adjustable loop and graft are passed through the tibial tunnel into the femoral tunnel (Figure 13A). Once the adjustable loop has fully traversed the lateral femoral cortex, it is flipped and tightened to secure the loop. The graft end is then tightened by applying reverse traction at the tibial side. Appropriate traction is maintained, and the knee joint is repeatedly flexed and extended 20 times within the range of motion. The graft is tightened on the tibial side with the knee flexed to 20° to 30°. Under traction, a guidewire is inserted next to the 4‐strand tendon graft, and a compression screw (Resorbable screw, Naton Company) is inserted along the direction of the guidewire. The compression screw is then screwed into the posterior lateral side of the graft. Next, a U‐shaped staple (MulFix U‐staple, Demey Company) is used for fixation. A final arthroscopic examination is performed to assess the graft tension and check for any impingement during knee flexion and extension (Figure 13B), and any debris is removed. Postoperative 3‐dimensional computed tomography images of the femoral tunnel are shown in Figure 14, with the overall schematic in Figure 15. The complete surgical procedure and technical details are shown in Video 1.

**FIGURE 13:**
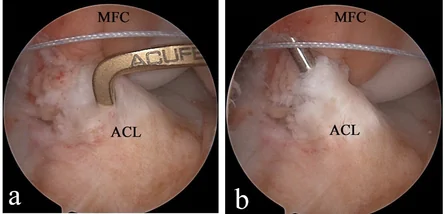
Creating the tibial tunnel as viewed arthroscopically through the anterolateral portal of the right knee. (A) Through the anterolateral portal, the knee joint is examined arthroscopically to identify the tibial footprint of the ACL. The tibial tunnel entry point is marked with a radiofrequency device, and the tibial tunnel guide is anchored at the center of the ACL remnant through the anteromedial incision. (B) The tibial guidewire is drilled through the guide, and a hollow reamer of the appropriate size is selected based on the graft diameter. The reamer is advanced along the guidewire to prepare the tibial tunnel. (ACL, anterior cruciate ligament; MFC, medial femoral condyle.)

**FIGURE 14:**
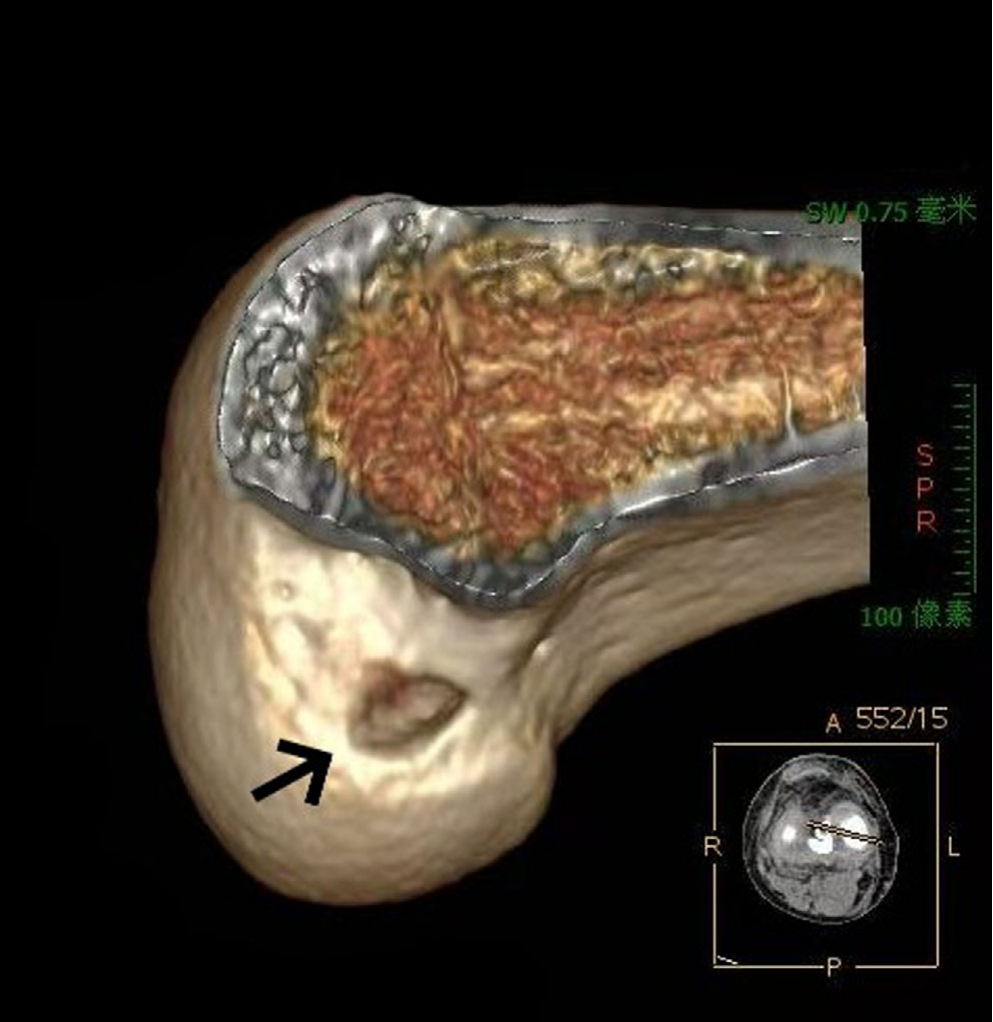
Postoperative 3‐dimensional computed tomography reconstruction of the right knee, medial view of the lateral femoral condyle. The image shows the precise placement and morphology of the oval femoral tunnel (black arrow), which effectively covers the anatomical footprint of the native anterior cruciate ligament.

**FIGURE 15:**
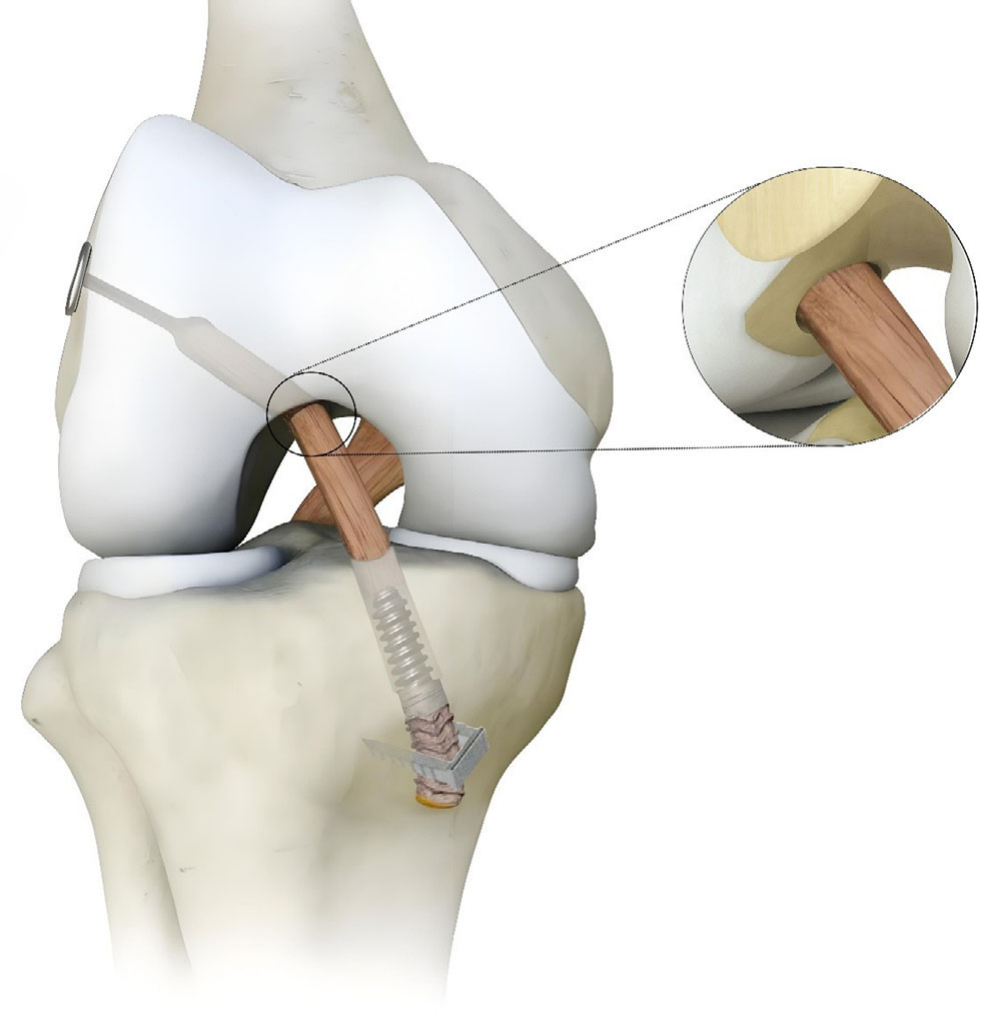
Overall schematic diagram of the fixation devices used for the oval tunnel. The graft was fixed on the femoral side with an adjustable‐loop suspensory cortical button. On the tibial side, an interference screw was inserted posterolateral to the graft, and fixation was reinforced externally with a staple to compress the graft.

## DISCUSSION

Before using our oval tunnel dilator, the position and orientation of the oval tunnel should be carefully planned based on preoperative magnetic resonance imaging and 3‐dimensional computed tomography to achieve optimal matching with the anatomical femoral footprint of the ACL. During surgery, fluoroscopy or 3‐dimensional computed tomography can be used to confirm tunnel placement and the long‐axis orientation of the oval dilator, as misalignment of the long axis may compromise the accuracy of anatomical restoration. When inserting the oval dilator, the knee should be placed in maximal flexion to avoid insufficient tunnel depth, which could adversely affect graft fixation. Femoral fixation is performed using an adjustable‐loop device, and no specific calculation of loop length is required. On the tibial side, the interference screw is placed on the posterolateral aspect of the graft, allowing the graft to assume a C‐shaped configuration that more closely conforms to the anatomical footprint of the ACL tibial insertion.^5^ Pearls and pitfalls of the oval femoral tunnel technique are described in Table 1.

**TABLE 1:** Pearls and Pitfalls of the Oval Femoral Tunnel Technique

Pearls	Pitfalls
Preoperative MRI planning to align tunnel direction with the ACL footprint	Since no pilot drill is used, forceful or off‐axis hammering of the expander can lead to lateral femoral condyle fractures
Gradual expansion with gentle, controlled impaction directly over the guidewire, not drilling, to preserve trabecular bone	Inadequate tunnel depth (<20 mm) may affect graft fixation
Use intraoperative fluoroscopy or 3D‐CT to confirm tunnel position	Misalignment of the long axis of the oval tunnel reduces anatomical restoration accuracy
The graft is fixed with an adjustable‐loop device and no calculation of loop length is required	The compression screw is placed posterolaterally to create a C‐shaped graft on the tibial side

3D‐CT, 3‐dimensional computed tomography; ACL, anterior cruciate ligament; MRI, magnetic resonance imaging.

The oval femoral tunnel technique offers several advantages and may serve as a feasible alternative for anatomical single‐bundle ACL reconstruction. First, the oval shape better matches the graft's natural shape, providing more uniform pressure distribution.^6^ For the same graft area, the oval shape has a longer circumference than the round shape, which results in a larger bone‐graft contact area, promoting biological healing.^7^ Second, according to geometric principles, grafts in oval tunnels are less likely to rotate compared with those in round tunnels, providing greater knee stability and promoting better graft integration. This minimizes graft instability and enhances tendon‐bone healing, leading to improved graft maturation. Our previous research using magnetic resonance imaging‐based signal‐to‐noise quotient evaluations showed that the oval tunnel group had lower values.^3^, ^4^ Finally, using an oval reamer to create the bone tunnel minimizes the risk of thermal damage to the tunnel walls and microfractures compared with a round tunnel created with a drill.^8^ Moreover, the impact‐expansion technique preserves the cancellous bone structure and further reduces the risk of tunnel enlargement.

However, this technique requires a higher level of technical skill, as precise anatomical positioning of the ACL is essential. If the surgeon is not sufficiently skilled at the outset, intraoperative fluoroscopy may be necessary, which could increase the overall surgical time. When using this technique to create the femoral tunnel, it is crucial to operate carefully and gently. Aggressive manipulation may result in tunnel fractures, and excessive expansion could compromise the fixation strength. The advantages and disadvantages of the oval femoral tunnel technique are summarized in Table 2.

**TABLE 2:** Advantages and Limitations of the Oval Femoral Tunnel Technique

Advantages	Limitations
Better anatomical restoration of the ACL footprint, enhancing knee stability	Requires specialized oval dilators, limiting widespread adoption
Larger bone‐graft interface promotes graft maturation	Tunnel positioning requires a learning curve
Lower risk of tunnel enlargement compared with round tunnels	Incorrect expansion can lead to intraoperative complications

ACL, anterior cruciate ligament.

## DECLARATION OF GENERATIVE AI AND AI‐ASSISTED TECHNOLOGIES IN THE WRITING PROCESS

During the preparation of this work, the authors used ChatGPT 5.2 to enhance the linguistic quality. After using this tool/service, the authors reviewed and edited the content as needed and take full responsibility for the content of the publication.

## DISCLOSURES

The authors (C.L., H.Z.) declare the following financial interests/personal relationships, which may be considered as potential competing interests: C.L. receives support (e.g., funding, provision of study materials, medical writing, and article processing charges) from Fujian Medical University Affiliated Union Hospital Research Project (no. 2024XH034). H.Z. receives support (e.g., funding, provision of study materials, medical writing, and article processing charges) from Fujian Medical University Affiliated Union Hospital Research Project (no. 2024XH032) and has been issued a patent (Chinese Patent issued No.: ZL201621452946.2). The other authors (W.L., C.W., Z.C., Z.W.) declare that they have no known competing financial interests or personal relationships that could have appeared to influence the work reported in this article.
